# Catalogue of National Medical Library

**Published:** 1876-06

**Authors:** 


Specimen Fasciculus of a Catalogue of the National Medical Library; under the Direction of the Surgeon General, United States Army, at Washington, D. C. Washington: Government Printing Office, 1876; 72 pp., 4to. *Aabec—Air.*

Of making many books there is no end indeed. The Surgeon General's Library, as it is usually called, contains 40,000

volumes and as many pamphlets, all carefully selected as to their bearing on medical science; and this, fearful to contemplate, is said to be only half of what a complete medical library should possess. The profession ought to feel thankful, however, that such valuable treasure is within the country; and, more, that it is likely to be made available—for an uncatalogued library is like an un-indexed book, of little practical value for reference.

The *Specimen* shows what may be done if Congress will give the money, and, as it stands, it is a marvel of completeness and convenience. This Library is especially rich in medical journals, which, as every one knows, contain the fresh facts and observations of the working men—the original material from which books are made.

By a lucid arrangement of cross references, every article in this vast collection of periodical literature, as well as in the more formal tomes, is carefully indexed under its appropriate head, and the student has in one group under his eye all that that thesaurus holds bearing on the subject. As an illustration of its magnitude, under the heading "Abdomen, Penetrating Wounds of," taken at random, are by actual count the titles of twenty-five distinct works and specific references to no less than 361 journal articles on the subject. The amount of labor that has been performed and the high order of intelligence necessary to its supervision, are simply wonderful. It is announced that the entire catalogue on this plan is nearly ready for the press, and we certainly hope that no parsimony, that may be mis-called economy, will prevent its suitable publication.

